# Requirements for swarming ability by lateral flagella on an agar surface in marine *Vibrio* cells

**DOI:** 10.1128/spectrum.02992-26

**Published:** 2026-10-15

**Authors:** Michio Homma, Takehiko Mima, Hajime Nakatani, Seiji Kojima

**Affiliations:** 1 Division of Material Science, Graduate School of Science, Nagoya University 12965, Nagoya, Japan; 2 Department of Medical Technology, Faculty of Health Sciences, Ehime Prefectural University of Health Sciences 84006, Iyo-gun, Japan; 3 Department of Biomolecular Engineering, Graduate School of Engineering, Nagoya University 12965, Nagoya, Japan; 4 Department of Biological Science, Graduate School of Science, Nagoya University 12965, Nagoya, Japan; University of Minnesota Twin Cities, St. Paul, Minnesota, USA

**Keywords:** motility, Laf, Pof

## Abstract

The marine bacterium *Vibrio alginolyticus* possesses two types of flagella in one cell: proton-driven lateral flagella extending from the periphery of the cell body and sodium ion-driven polar flagella extending from a cell pole. It is believed that, for swimming in seawater, they use polar flagella, suppressing the expression of lateral flagella, and when they attach to the body surface of a fish or the intestinal tract, lateral flagella are induced, allowing them to crawl along the surface or through mucus. The dynamometer hypothesis, which proposes that polar flagella sense rotation and control the expression of lateral flagellar genes, has been presented. However, how rotation is sensed and how expression is controlled remain unclear. Although swarming has recently been analyzed by physical, biological, or biochemical perspectives, it remains unclear how this motility is controlled, or which substances and conditions are necessary for swarming ability. In this study, we discovered that adding gelatin to agar medium promotes swarming on the agar surface by the lateral flagella of *Vibrio*. Based on our data, we speculate that surfactants or viscous polysaccharides secreted extracellularly are important for promoting swarming on the agar surface, and we identified that swarming is likely to be driven by S (social)-motility, in which bacteria move by interacting with each other, and A (adventure)-motility, in which bacteria move by interacting with the agar surface. Our study provides clues that help clarify the mechanism of bacterial swarming.

**IMPORTANCE:**

We discovered that adding gelatin to hard agar medium promoted swarming on agar surfaces by the lateral flagella of *Vibrio* cells. The surfactants or viscous polysaccharides secreted extracellularly seem to be important for swarming ability on agar surfaces. We proposed that the swarming is thought to occur through S (social)-motility, where cells move by interacting with cell bodies each other, and A (adventure)-motility, where cells move by interacting with the agar surface and cell body. The present study should provide clues to clarify the mechanism of bacterial swarming and how to move in a viscous environment.

## INTRODUCTION

Bacteria are known to swim in liquid media, but the ability to swarm on solid media or an agar plate was first identified in *Proteus* bacteria (1, 2). Subsequently, surface swarming was found to be widespread in flagellated bacteria (3, 4). Bacterial movement on surfaces such as agar can occur by multiple mechanisms, including the extension and retraction of specialized pili (“twitching”) (5, 6), the movement of membrane-bound adhesins along the cell surface (“gliding”) (7, 8), a detergent-assisted but primarily passive growth mechanism (“sliding”) (9), or swimming in a thin layer of fluid at the surface (“swarming”) (10, 11). Sliding and swarming on agar surfaces have been observed in a variety of genera, including *Proteus* (1), *Vibrio* (12, 13), and *Bacillus* (14), as well as the gram-negative model species *Salmonella enterica* and *Escherichia coli* (15). *S. enterica* and *E. coli* require relatively low agar concentrations (approximately 0.5%) for migration on the agar surface (15, 16). In contrast, *Proteus mirabilis* can migrate on harder surfaces (>2% agar) (17). While swarming of *Bacillus subtilis*, *Staphylococcus aureus*, and *Pseudomonas aeruginosa* depends on cell-produced surfactants, swarming of *E. coli* and *S. enterica* is thought to rely on cell-derived osmotic agents that draw water from the medium and form a wet zone around the edge of the expanding colony (11, 18–21). However, the osmotic agents have not been identified. Flagella-deficient and motility-deficient strains do not swarm, indicating that cell migration within this fluid layer depends on active propulsion by flagella.

The flagellar motor consists of a rotor and surrounding stator subunits (22–25). The stators are ion channels that convert the electrochemical potential energy of protons or sodium ions across the cell membrane into kinetic energy. The rotor comprises an MS ring embedded in the cell membrane and a cytoplasmic C-ring attached beneath the MS ring. The stator is composed of two types of membrane proteins, the A subunit and the B subunit (26). For example, MotA and MotB form the proton-driven stator in *E. coli*, PomA and PomB form the sodium-driven stator in *Vibrio*, or MotP and MotS form the sodium-driven stator in *Bacillus*. The A subunit (MotA/PomA/MotP) comprises four transmembrane helices and a large cytoplasmic domain, which contains highly conserved charged residues essential for torque generation and interacts with the C-ring component protein FliG to drive the rotor rotation. The B subunit (MotB/PomB/MotS) consists of a single transmembrane helix and a C-terminal OmpA-like domain. This transmembrane helix contains conserved aspartic acid residues essential for the ion channel activity of the stator. The OmpA-like domain binds to the peptidoglycan layer or T-ring, anchoring the stator complexes around the rotor (27).

The marine *Vibrio* (*V. alginolyticus*) possesses two types of flagella in one cell: proton-driven lateral flagella (Laf) located around the periphery of the cell and sodium ion-driven polar flagella (Pof) located at the cell poles (28, 29). When swimming in seawater, they use polar flagella, and the lateral flagella are suppressed. It is believed that, in nature, the lateral flagella are induced when attached to the surface of a fish or the intestinal tract, allowing them to crawl along the surface or through mucus (30, 31). Expression of the lateral flagella is also induced by amiloride derivatives, sodium channel inhibitors, that inhibit sodium-driven polar flagellar motility (32). These observations led to the proposal of the widely accepted dynamometer hypothesis, which states that flagella can sense rotation and control flagellar gene expression (33, 34). However, how rotation is sensed and how expression is regulated remain unclear. Motility via lateral flagella is possible on the agar surface of hard agar plates (1.25%), but not on the agar surfaces of 2.5% or higher agar (10, 35). This phenomenon of crawling on the agar surface has also not been fully explained.

FliL has been identified as a protein that assists stator function (36). *V. alginolyticus* has two types of FliL for polar and lateral flagella. Mutants of FliL (^pof^*fliL* and ^laf^*fliL*) exhibit reduced polar flagellar motility at high viscosity and are defective in lateral flagellar motility on hard agar plates, respectively (37). Therefore, FliL is closely involved in the swarming ability of lateral flagella. FliL is a single-pass transmembrane protein reported to be located near the stator and basal body, interacting with them (38, 39). It has also been shown that FliL is important for the efficient incorporation of the stator into the motor (38). A possible model has been proposed that FliL interacts with the stator to form a stator-FliL complex surrounding the stator (37). Structural analysis revealed that FliL has a stomatin/prohibitin/flotillin/HflK/C (SPFH) domain, which is widely conserved from mammals to bacteria. The SPFH domain is also known to be involved in mechanosensing and ion channel regulation (40, 41).

Surface swarming by flagella has recently been analyzed from a physics point of view (42, 43). However, from the biological or biochemical perspective, it is still unclear how the swarming motility is regulated and what materials or conditions are necessary for the swarming ability. In this study, we found that the addition of gelatin in hard agar plates extremely promotes swarming never observed before on the surface of agar by marine *Vibrio* lateral flagella. We suggest that surfactants or viscous polysaccharides secreted outside the bacterial cell are important for the swarming motility on the agar surface.

## RESULTS

### Swarming ability induced by gelatin

**Fig 1:**
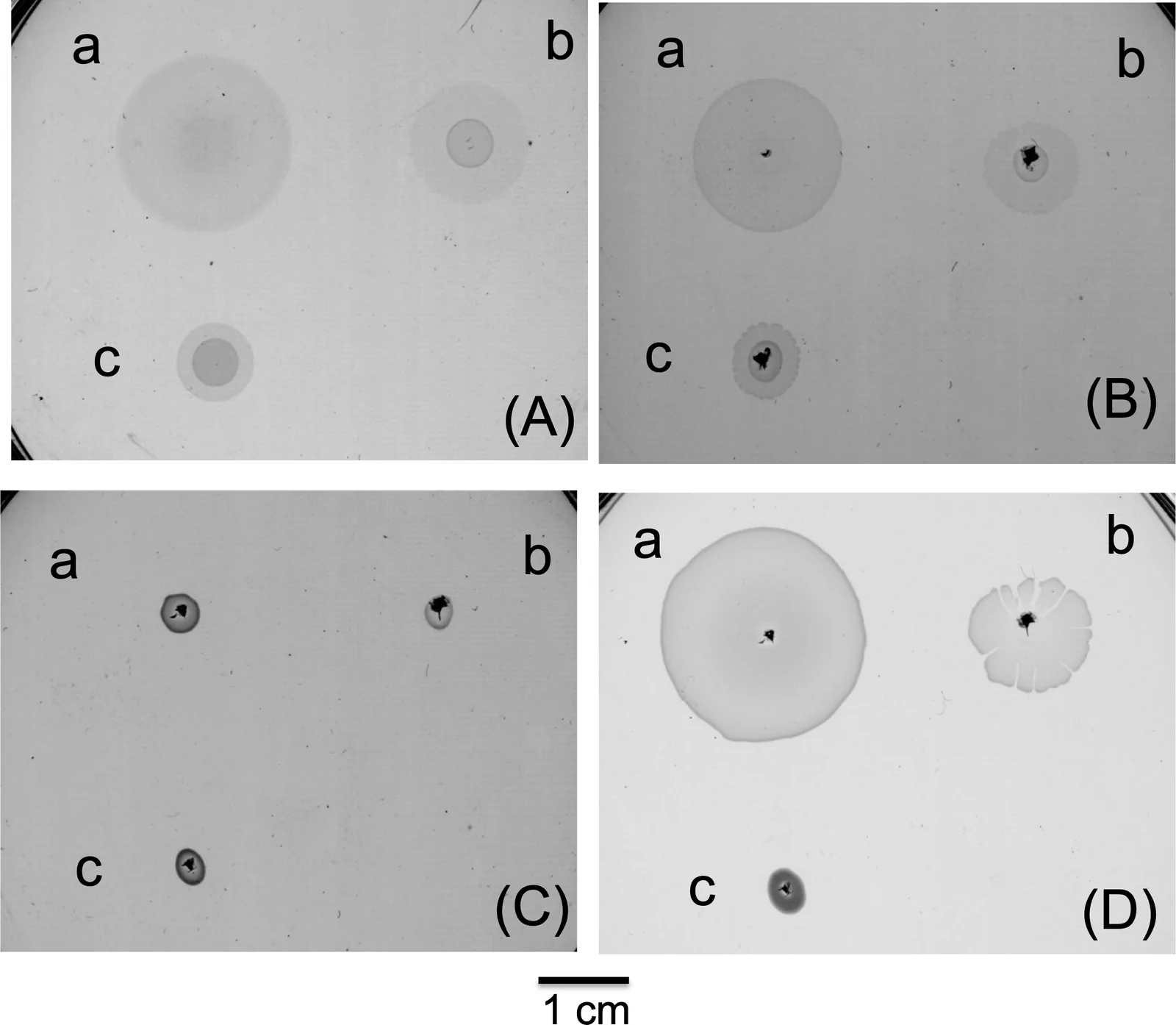
Swarming by gelatin addition. Two microliters of the overnight culture (a: 138-2, b: YM19, and c: YM19Δ^laf^fliL) was applied to the 1.25% agar-VNG plate containing 1% gelatin (**A**), 1% collagen peptides (**B**), 1% casamino acid (**C**), and incubated at 30°C. The plates were scanned with a photo scanner after 6 h (**A–C**) and the plate (**C**) was further incubated for 10 h (**D**).

**Fig 2:**
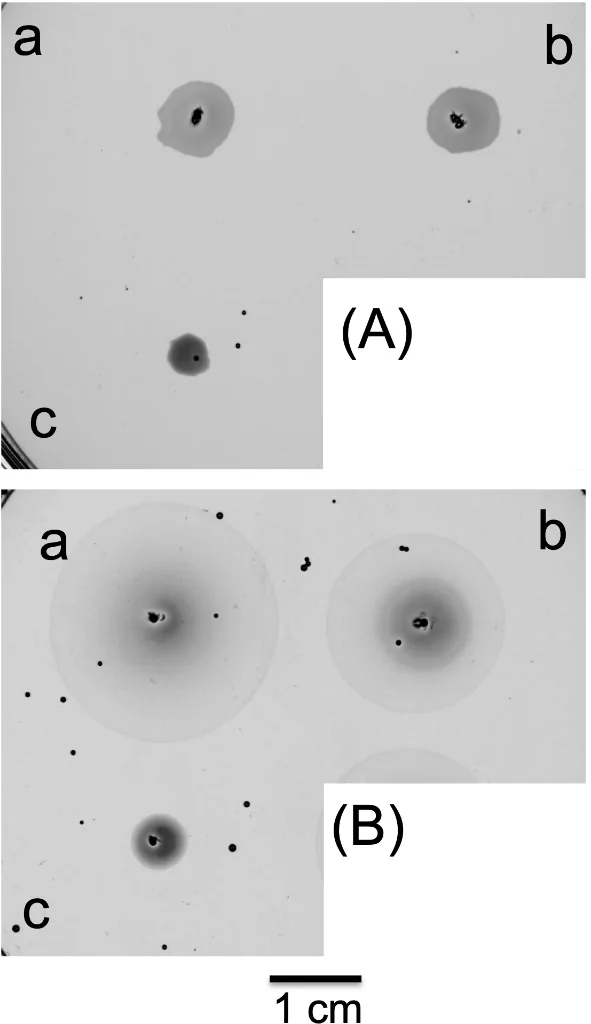
Swarming by PVP addition. Two microliters of the overnight culture (a: 138-2, b: YM19, and c: YM19Δ^laf^fliL) was put on the 1.25% agar-VNG plate (**A**), or containing 5% PVP (**B**). The plates were scanned with a photo scanner after 6 h.

The motility of *Vibrio alginolyticu*s has been previously analyzed using polar and lateral flagellar mutants (29). In that report, the motility on agar plates was examined using heart infusion broth (Difco) containing 1.5% agar. Moreover, it was shown that lateral flagellar-deficient mutants prevented motility on 1.5% agar, even though polar flagella are present. Lateral flagella were also shown to be capable of motility in highly viscous environments containing polyvinylpyrrolidone (PVP), a water-soluble polymer that confers high viscosity to the buffer or medium (30). Here, we discovered the medium conditions required for swarming motility on 1.25% agar for the first time with the reproducible swarming profiles (Fig. 1). Overnight cultures of the wild-type strain (138-2), the polar flagella (*pof*)-deficient strain (YM19), and the polar flagella and lateral *fliL* (*pof* ^laf^*fliL*)-deficient strain (YM19Δ*fliL*, named NMB342) were spotted onto 1.25% agar VNG plate and incubated at 30°C for 6 h. The swarming ring formation by bacterial spreading was limited on media containing only casein hydrolysate as a nutrient source (Fig. 1C), but was significantly greater on media containing bovine gelatin (Fig. 1A). The ^laf^*fliL* mutant (shown as c in Fig. 1) showed very limited spreading. After extended incubation, even in the agar plate without gelatin, the bacterial cells, except the ^laf^*fliL* mutant, were able to spread on the 1.25% agar (Fig. 1D). This suggested that the addition of gelatin induced the release of products that enabled spreading of the swarming ring on the agar surface. The induction of swarming ability also occurred with the addition of chitin oligosaccharides and collagen peptides, the hydrolyzed products of bovine or fish gelatin (Fig. S1). Furthermore, adding PVP (final concentration of 5%) to the 1.25% agar medium enabled bacterial spreading on the agar surface, similar to profiles by adding gelatin (Fig. 2). The ^laf^*fliL* mutant did not induce swarming by PVP. We hypothesized that gelatin may induce the secretion of a highly viscous substance such as PVP outside the cell.

Next, we observed the flagellar formation of swarming cells on the agar plates. After overnight cultures were spotted onto the 1.25% agar VPG plate and incubated at 30°C for 6 h, the edges of the colonies were observed using an electron microscope (Fig. S2). In the wild-type strain (138-2), numerous lateral flagella were observed around the cell periphery, whereas the polar flagellum was not observed or distinguished under the present conditions. In the *pof*-deficient strain (YM19), numerous lateral flagella were observed around the cell periphery as in wild-type cells, but the polar flagellum was not observed. In the *pof* ^laf^*fliL*-deficient strain (NMB342), numerous lateral flagella were observed around the cell periphery, although the number of lateral flagella seems to be slightly lower than in the *pof*-deficient parent strain YM19.

**Fig 3:**
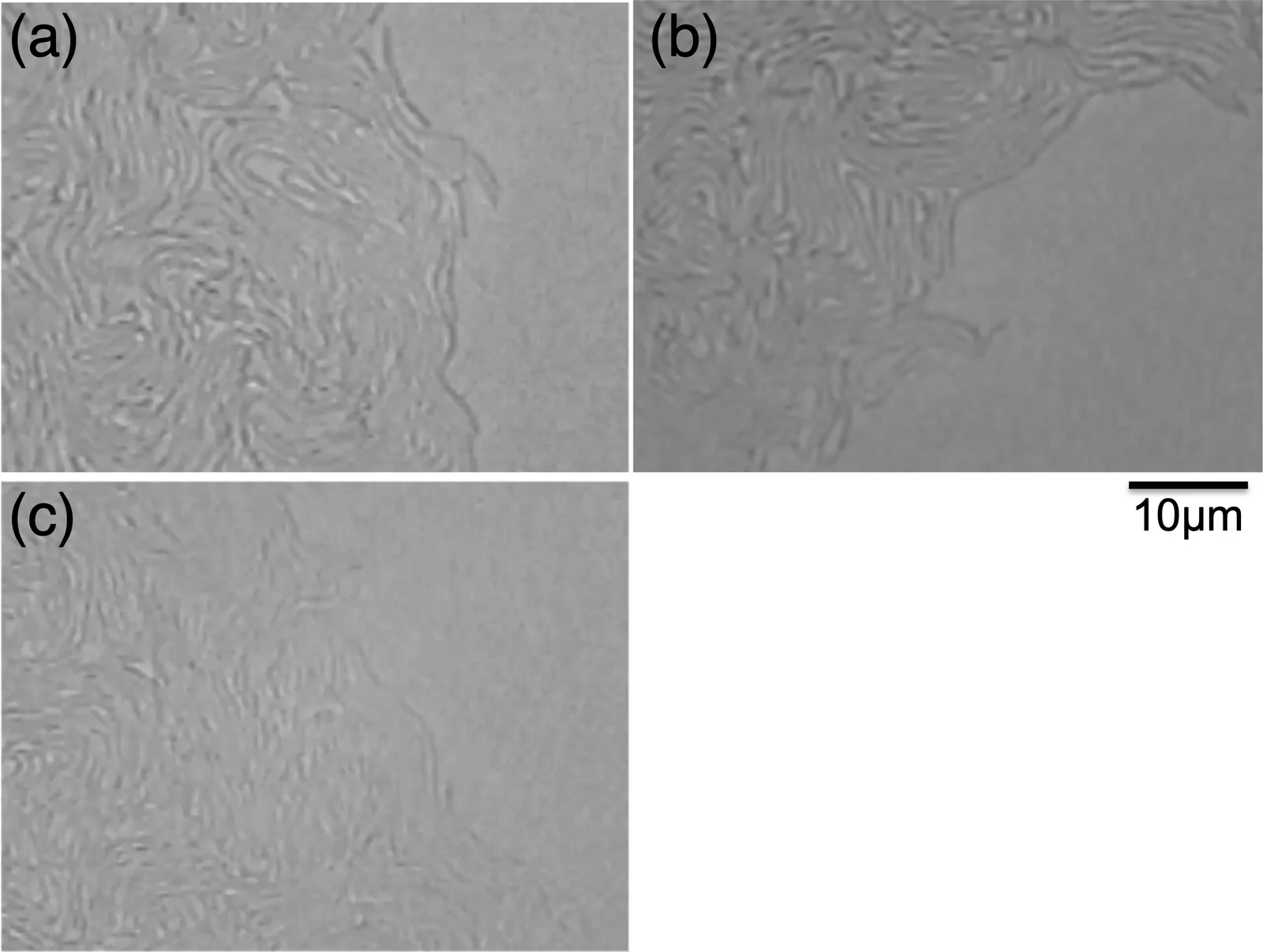
Swarming profiles of cells on an agar plate. Two microliters of the overnight culture (a: 138-2, b: YM19, and c: YM19^laf^*fliL*) was inoculated and incubated at 30°C for 6 h on the 1.25% agar-VNG plate containing 1% gelatin. The edges of swarming rings (for 138-2 and YM19) or the colony (YM19^laf^*fliL*) were observed by digital microscope (see also Fig. S3).

We directly observed cell motility on the agar. After overnight cultures were spotted onto 1.25% agar and incubated at 30°C for 6 h, cells at the edges of the colonies were observed using a digital microscope (Fig. 3; Fig. S3, movies that demonstrate motility at the edge are included). On the gelatin-containing agar medium, the wild-type and *pof*-deficient strains, whose cells were elongated and in a mostly single layer, moved through a wriggling motion (Fig. S3a and b). The *pof* ^laf^*fliL*-deficient strain moved very slowly, and the cells were multi-layered due to their inability to move outward (Fig. S3c). Areas other than the edge of the swarming ring of the *pof*-deficient strain on 1.25% agar medium were also observed using digital microscope video (Fig. S4). The size and motility of the cells are different among the places of the ring. Other than the edge regions, bacterial cells were multi-layered, elongated, and moving everywhere in all locations. Cells of the inner region appear less elongated because the cells had been divided.

### Swimming ability in the presence of PVP

**Fig 4:**
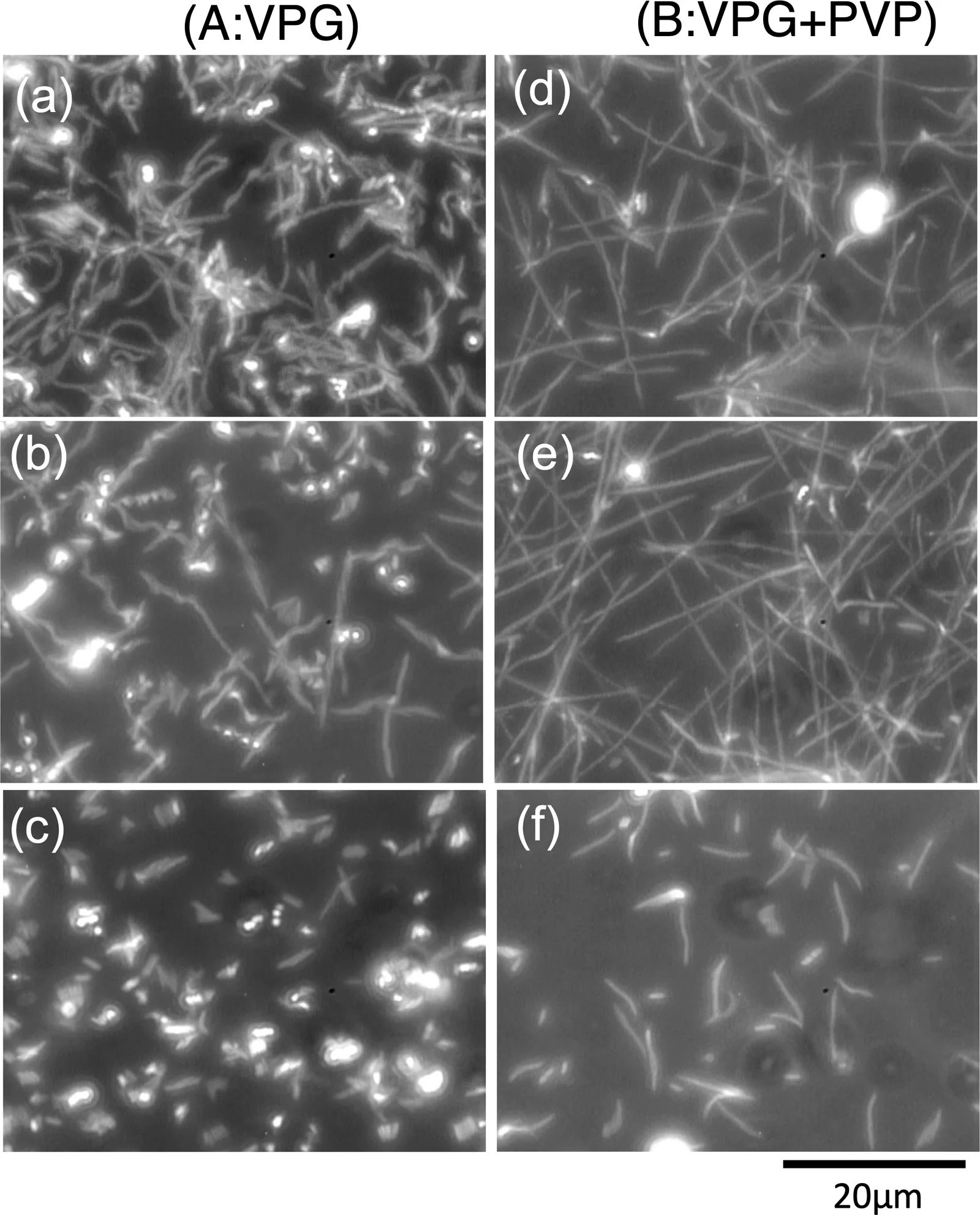
Swimming motility in a culture with or without PVP. Fifteen microliters of the overnight culture (a, d: 138-2; b, e: YM19; and c, f: YM19Δ^laf^fliL) was inoculated into 1 mL of VPG (**A**) or VPG containing 5% PVP (**B**) and incubated at 30°C for 3 h. The cells were observed with a cover glass by dark-field microscopy and recorded by a CCD camera. The 30 images (1 s) were integrated using motion analysis software.

It has previously been shown that the viscosity of the medium significantly affects the swimming motility of *V. alginolyticus* cells (30). Here, in wild-type cells, which possess both polar and lateral flagella, only the polar flagella were constitutively expressed in low-viscosity media, resulting in forward and backward swimming with a characteristic phenotype by polar flagella (Fig. 4a; Fig. S5a). On the other hand, in high-viscosity media containing PVP, the lateral flagella were expressed and the bacteria swam smoothly (Fig. 4d; Fig. S5d). However, in the *pof*-deficient strain (YM19), the lateral flagella were expressed even in low-viscosity media, resulting in motility with reduced frequency of directional changes (Fig. 4b; Fig. S5b). By contrast, the *pof* ^laf^*fliL*-deficient strain (NMB342) was almost completely non-motile in low-viscosity media, but showed enhanced motility in high-viscosity media (Fig. 4c and f; Fig. S5c and f). This evidence may contradict the previous reports suggesting that FliL is essential for resisting the stress by high viscosity. This contradiction may be derived from the variable functions of FliL that have been reported among the various species or the functions of FliL for the flagellar motor between polar flagella and lateral flagella are different (36–39).

### Swarming and swimming ability of *fliL* mutant

**Fig 5:**
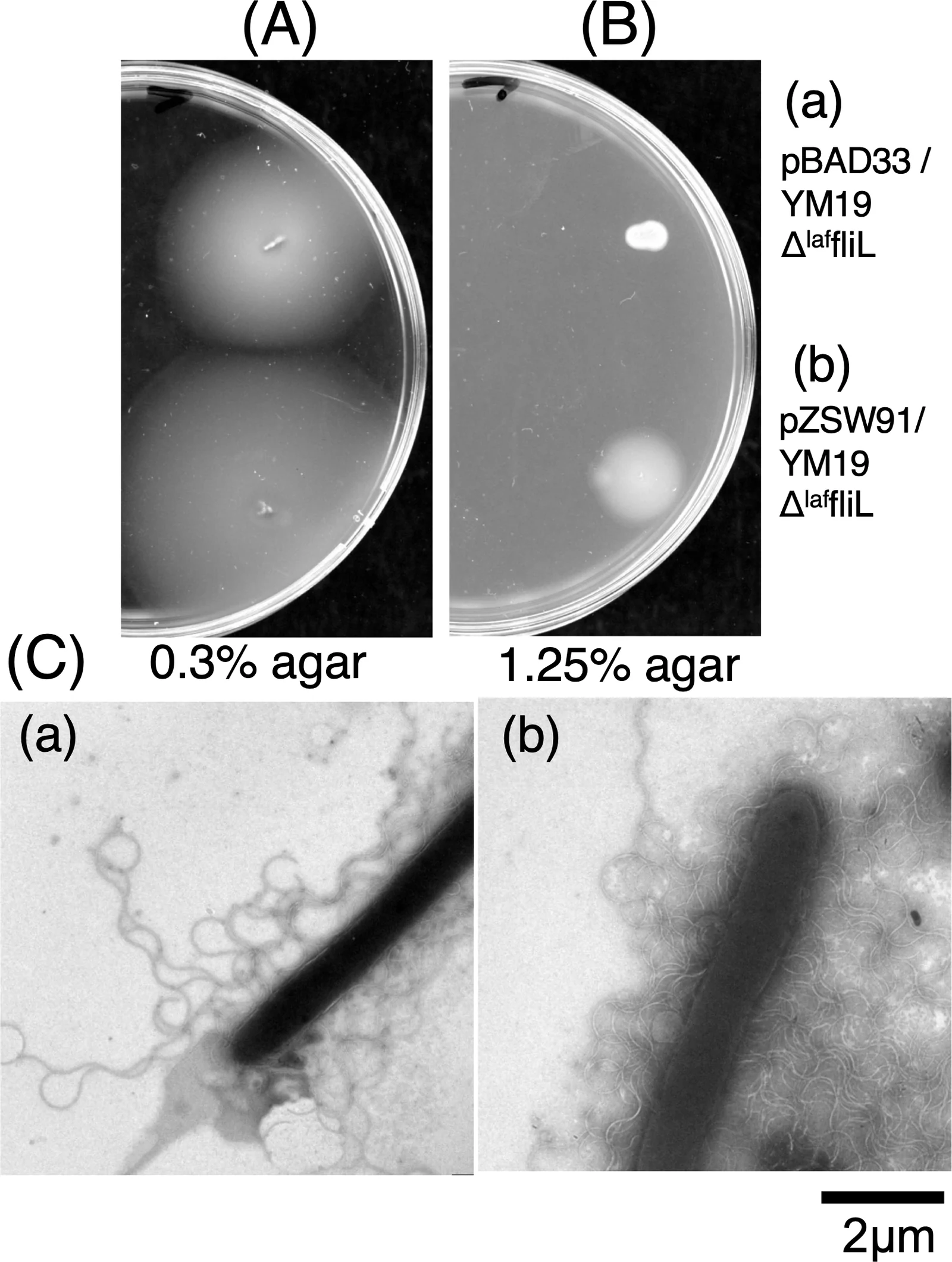
Effect of *fliL* gene on motility. Two microliters of the overnight culture of YM19Δ^laf^*fliL* harboring pBAD33 (vector) (a) and pZSW91 (^laf^*fliL*) (b) was applied to the 0.3% agar-VPG plate (**A**) or 1.25% agar-VPG plate (**B**), containing 2.5 μg/mL Cm and 0.1% arabinose and incubated at 30°C. The plates were scanned with a photo scanner after 24 h. The edges of the colonies from panel **B** were picked up with a toothpick, suspended in 1% PTA, and observed under an electron microscope (**C**).

We examined the function of ^laf^*fliL* using the *pof* mutant cells (Fig. 5). On 1.25% agar, the swarming ring of the ^laf^*fliL* mutant did not expand, but the mutant cells with the ^laf^*fliL* gene introduced had an expanded swarming ring. On 0.3% agar, the swimming ring of the ^laf^*fliL* mutant was slightly smaller than that of the mutant cells with the ^laf^*fliL* gene, although they exhibited good motility. This confirmed that the ^laf^*fliL* mutation plays a major role in the swarming ability on the agar surface. Digital microscope video observation of the edge of the swimming ring revealed that the ^laf^*fliL* mutant and the ^laf^*fliL* mutant complemented by the ^laf^*fliL* gene gave almost identical swimming motility in soft agar, confirming that the ^laf^*fliL* gene does not contribute to the swimming ability (Fig. S6).

### Extracellular materials of the swarming cells

**Fig 6:**
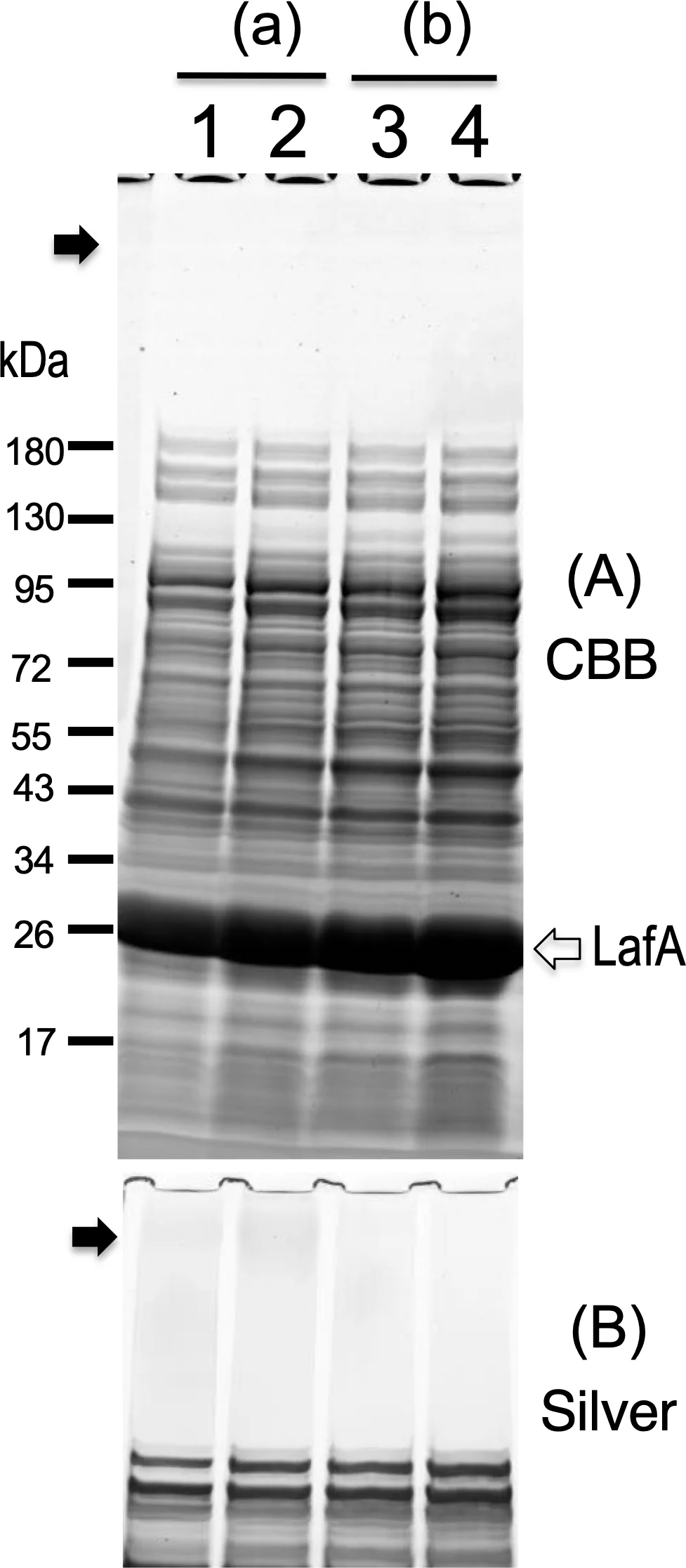
SDS-PAGE profiles of cell surface materials. Fifty microliters of overnight culture (a)YM19 (1, 2) and (b)YM19Δ^laf^*fliL* (3, 4) were inoculated onto 1.25% agar-VNG plates (without gelatin: 1, 3 and +1% gel: 2, 4), and incubated at 30°C. After 6 h, the cells were scraped and suspended in 1 mL of 20TN200. The cells were stirred using a homogenizer and removed by low-speed centrifugation. The supernatant was collected as the sample and was separated by SDS-PAGE, stained with Coomassie Brilliant Blue (**A**: CBB) and silver (**B**: silver), and photographed.

The cells grown on 1.25% agar medium were harvested and homogenized to obtain extracellular materials. The separated materials were detected by SDS-PAGE, followed by CBB staining and silver staining (Fig. 6). The most abundant protein in the extracellular materials seems to be flagellin (LafA), indicated by an open arrow in Fig. 6A. The staining profiles are similar either in the presence or absence of gelatin in the low-molecular weight region, though the high-molecular-weight region (indicated by closed arrows) stained by silver in the presence of gelatin is denser than that in the absence of gelatin. This might indicate that the size of extracellular pos is increased by the addition of gelatin.

## DISCUSSION

Previous studies have shown that swarming on agar in various bacteria is significantly affected by various factors such as temperature, salt concentration, and agar concentration. In this study, we found that the swarming ability on the agar surface by the lateral flagella of *V. alginolyticus* is significantly affected by the addition of gelatin. The swarming was also promoted by the addition of gelatin degradation products (collagen peptides) or a chitin degradation product, although the swarming profiles are slightly different. The swarming edge was distorted when fish collagen peptides were included in the plate (Fig. S1c), and the lack of ^laf^*fliL* affected swarming more severely when chitin was included in the plate (Fig. S1a). We do not know the actual reason for the difference; however, we speculate that the additives on the plate would affect somewhat differently the expression of flagellar genes or genes that support the swarming, such as the polysaccharide synthesis genes. Furthermore, the addition of the viscous substance PVP also promoted swarming without gelatin. These findings imply that the secretion of viscous substances from the bacterial cell is necessary for the swarming promotion on the agar surface. Factors involved in biofilm formation are thought to influence the production of extracellular materials and affect swarming or swimming by flagella (44, 45). Surfactant secretion could also be a factor promoting swarming. It has been reported that the addition of Tween-80 to agar medium promotes the formation of swarming rings (46). Comparison of extracellular substances from cells grown in the absence and presence of gelatin, a swarming-promoting factor, revealed almost no significant difference in proteins stained by CBB or polysaccharides stained by silver staining, though polysaccharides of high molecular weight seem to be increased slightly. We plan to conduct a more detailed analysis of the extracellular substances in the future.

**Fig 7:**
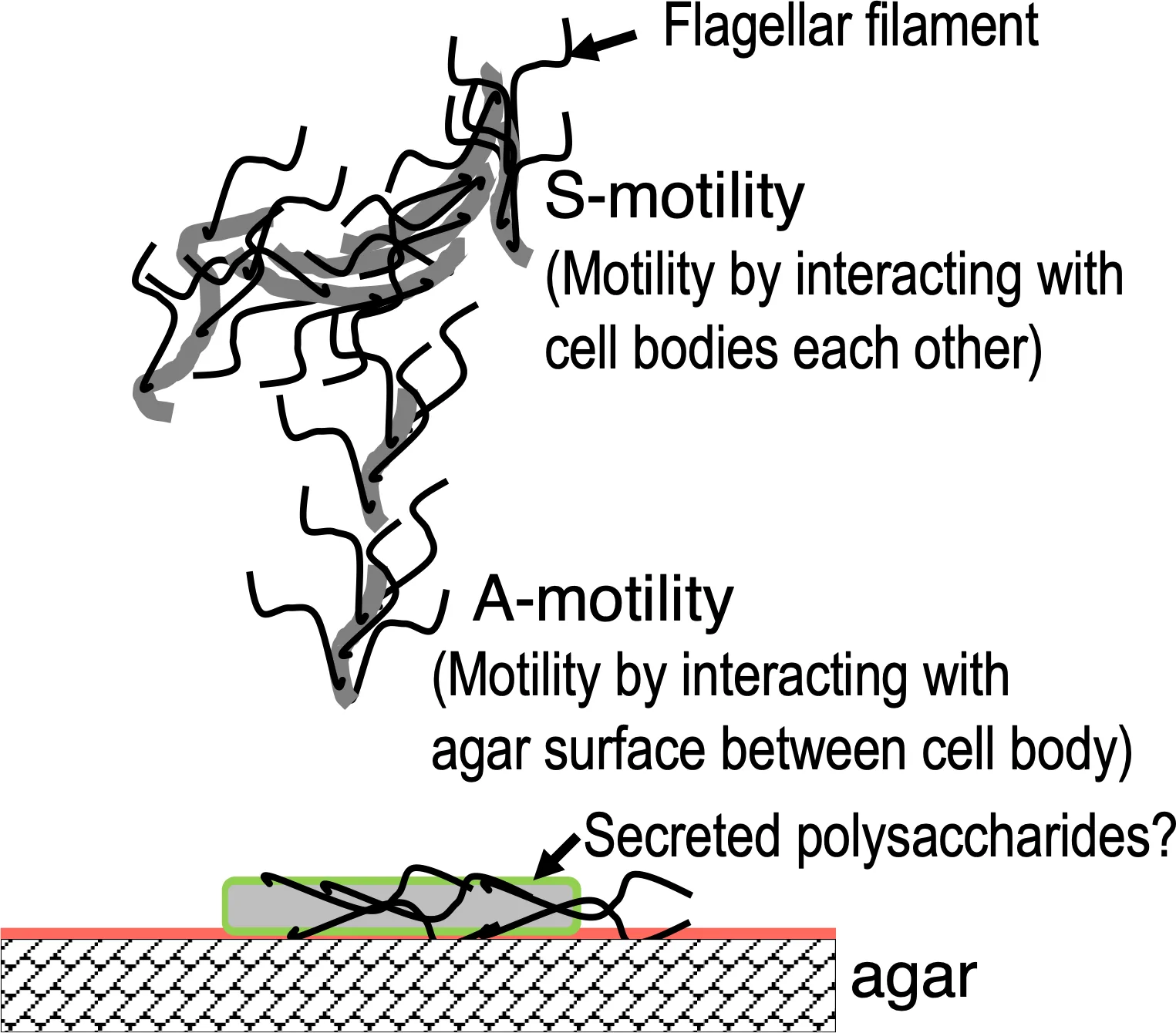
Model of cell motility by lateral flagella on agar. The water layer on agar and the cell surface layer are shown as the orange line and the green line, respectively. The cells and flagellar filaments are shown by gray lines and black lines, respectively.

From the observations of the movement of colony cells, there appear to be two ways in which the propulsive force is generated: (i) interactions between the cell surface and the agar surface, and (ii) surface interactions between cells themselves (Fig. 7). We can imagine that the flagellar filament is the tire of a car, and the agar surface is the road surface. These two kinds of movements phenotypically resemble the motility of *Myxococcus*. When a single cell or a small group of *Myxococcus* cells move, they exhibit A (adventure)-motility, whereas when they move as a large group, they show different motility, called S (social)-motility (47, 48). It has been proposed that the swarming motility of *S. enterica* and *E. coli* is driven by flagella swarming through the water on the agar surface (49). In our observations in this study, vigorous cell movements by the interaction with elongated cells did not promote the ring formation or the swarming on agar plates. It is likely that interactions on the cell surface are responsible for the motility. Flagella, which are locomotive machines, are located in the outermost part of the cell, and we could imagine that flagella interact with each other to drive motility. Interactions between the agar surface and the cell surface or flagella seem to promote rapid spreading on the agar surface (A-motility), forming a single-cell layer that spreads outward from the place where the cells were spotted. Depending on the conditions of the agar surface and/or of the cell surface, the driving force is not generated between the cell surface and the agar surface; thus, A-motility does not occur, and it is possible to spread the swarming ring formed by movements of the cell-cell interactions via S-motility. The expansion of the cell ring due to S-motility is slower than that due to A-motility, and the cells spread in multiple layers.

It has been shown that the expression of lateral flagella is required for cell migration on plates (50). In the wild-type strain (138-2), lateral flagellar expression is suppressed in liquid medium, and cell movement is achieved by constitutively expressed polar flagella (29). When cells are plated on a 1.25% agar surface, lateral flagella are expressed, enabling the cell to swarm on the agar surface. Regulation of lateral flagellar expression is similar to that of biofilm formation, and c-di-GMP signaling is involved in the regulation (51) (Fig. S7). Gene expression during gelatin-stimulated swarming is thought to be a very complex process, involving the c-di-GMP regulation network. So far, there have been no insights into how gelatin induces the swarming motility except for the finding that gelatin is involved in the VarS/VarA two-component regulatory system, which regulates the expression of collagenases that degrade gelatin (52). The homologous GacS/GacA system in *P. aeruginosa* has been shown to be involved in the regulation of flagellar formation and biofilm formation (53). We speculate that the VarS/VarA system is involved in the production and/or secretion of viscous materials or biosurfactants. *V. alginolyticus* and its relatives are important marine decomposers and are involved in chitin degradation. We showed here that chitin oligosaccharides also stimulated swarming (Fig. S1). From the evidence, it is possible that the induction of the degradation systems and the production of swarm-promoting substances, possibly surfactants or viscous polysaccharides, might use the same regulation pathway. It is noteworthy that a defect in ^laf^FliL prevents swarming but not swimming in agar. The swimming of the ^laf^*fliL* mutant cells in liquid medium was inhibited compared to the wild-type cells and was promoted by the addition of PVP, which increases the surrounding viscosity of the cells. The *fliL* protein may affect the gene expression for swarming, as shown above (Fig. S7). The supplementary effects to promote the swarming seem to be too complicated. However, we hope to identify the swarming-promoting factors and how the environmental signals are sensed and the production is regulated in the future.

## MATERIALS AND METHODS

### Strains, plasmids, and media

The bacterial strains and plasmids used here are listed in Table S1. *V. alginolyticus* cells were cultured at 30°C in VC medium (0.5% polypeptone, 0.5% yeast extract, 0.4% K_2_HPO_4_, 3% NaCl, and 0.2% glucose), VPG medium (1% polypeptone, 0.4% K_2_HPO_4_, 3% NaCl, and 0.5% glycerol), or VNG medium (1% NZ amine, 0.4% K_2_HPO_4_, 3% NaCl, and 0.5% glycerol). When necessary, chloramphenicol was added to final concentrations of 2.5 µg/mL for *V. alginolyticus*, after media had been autoclaved. Bacterial cells were suspended in 20TN200 (20 mM Tris-HCl, pH 8.0, containing 200 mM NaCl).

### Expansion assay of cells on agar plates

VPG or VNG-0.3 soft agar plates (0.3% agar), VPG or VNG-1.25 hard agar plates (1.25% agar), or VPG or VNG-2.5 hard agar plates (2.5% agar) were used for *V. alginolyticus* expansion assay. When necessary, gelatin from bovine bone (Fujifilm, Japan), casamino acids (BD, USA), collagen peptides (Nestle, Japan), or fish collagen peptides TFF-01 (Nippi, Japan) and polyvinylpyrrolidone PVP K90 (TCI, Japan) were added to final concentrations of 1% and 5%, respectively, and autoclaved. An aliquot (2 µL) of overnight culture was spotted onto a plate, which was then incubated at 30°C in a wetted container. The size of the ring expansion was recorded by a scanner. The expansion ring of cells was observed by stereomicroscope (VHX2000, Keyence Co., Osaka, Japan) equipped with a high-magnification zoom lens (VH-Z450, Keyence Co.) at 3,000-fold magnification. Movies were recorded on a computer (Mac mini) using USB video capture. Those movies are included in the Fig. S3, S4, and S6. Only representative data are shown in Fig. 1 and 2, and data reproducibility was confirmed by multiple experiments using biological replicates.

### Observation of swimming behavior

*V. alginolyticus* cells were cultured overnight in VC medium at 30°C. The overnight culture was suspended in VPG medium at 50- or 100-fold dilution and incubated at 30°C for 6 h, and cells were observed under a dark-field microscope and recorded by a CCD camera on a computer or by a digital camera. Swimming profiles were analyzed using motion analysis software (DIPP-Motion V, DITECT, Japan).

### Transmission electron microscopy

The cells of *V. alginolyticus* were picked from the agar plates and mixed with 1% (wt/vol) potassium phosphotungstate solution (pH 7). The solution was placed on the carbon-coated copper grid and observed with an electron microscope (JEM-1400, JEOL, Japan).

### SDS-PAGE analysis of the cell surface materials

Overnight cultures of YM19 and YM19Δ*fliL* were inoculated onto 1.25% agar-VNG plates with or without 1% gelatin, and incubated at 30°C. After 6 h, the cells were scraped and suspended in 1 mL of 20TN200. Cell suspensions were stirred using a Polytron for 30 s twice, and centrifuged at 10,000 rpm for 15 min. The supernatant was collected as the samples and was separated by SDS-PAGE, stained with Coomassie Brilliant Blue or silver, and photographed.

## Data Availability

The data that support the findings of this study are available in the Supplemental material of this article.

## SUPPLEMENTAL MATERIAL

Figure S1 spectrum.02992-26-s0001.pptx Swarming profiles of Vibrio cells on 1.25% agar containing various supplements. 2 μL of the overnight culture (a:138-2, b:YM19, c:YM19Δ^laf^fliL) was put on the 1.25% agar-VNG plate containing 1% chitin oligosaccharides (A), 1% beef collagen peptides (B), 1% fish collagen peptides (C) or none (D), and incubated at 30°C for 5 hours. The plates were scanned with a photo scanner.

Figure S2 spectrum.02992-26-s0002.pptx Flagella formation observed by electron microscopy. 2 μL of the overnight culture of 138-2 (a), YM19 (b), or YM19Δ^laf^fliL (c) was put on the 1.25% agar-VPG plate and incubated at 30°C overnight. The edges of the colonies were picked up with a toothpick, suspended in 1% PTA, and observed under an electron microscope. Bar shows 2μm.

Figure S3 spectrum.02992-26-s0003.pptx Movement of *Vibrio* cells on 1.25% agar containing gelatin. 2 μL of the overnight culture (a:138-2, b:YM19, c:YM19Δ^laf^fliL) was inoculated on the 1.25% agar-VNG plate containing 1% gelatin and incubated at 30°C for 6 hours. The edges of the colonies were observed using a digital microscope.

Figure S4 spectrum.02992-26-s0004.pptx Movement of YM19 cells at the various positions of the swarm ring. 2 μL of the overnight culture of YM19 was put on the 1.25% agar-VNG plate containing 1% gelatin and incubated at 30°C. The plates were scanned with a photo scanner after 6 hours (f) and the various places (a-e) of the colony were observed using a digital microscope.

Figure S5 spectrum.02992-26-s0005.pptx Swimming profiles of cells in medium with or without PVP. 10μL of the overnight culture (a, d:138-2, b, e:YM19, c, f:YM19Δ^laf^fliL) was inoculated into 1mL of VPG (A) or VPG containing 5% PVP (B) and incubated at 30°C for 6 hours. The cells were observed with a cover glass by phase contrast microscopy using a 40X objective lens.

Figure S6 spectrum.02992-26-s0006.pptx Swimming of the *fliL* mutant in 0.3% agar. 2 μL of the overnight culture of YM19Δ^laf^fliL harboring pBAD33 (a) and pZSW91 (b) was put on the 0.3 % agar-VPG plate containing 2.5μg/mL Cm and 0.1% Arabinose and incubated at 30°C for 8 hours. The edges of the colonies were observed using a digital microscope.

Figure S7 spectrum.02992-26-s0007.pptx Possible regulation of lateral flagella, polar flagella, biofilm, or collagenase in *Vibrio* cells. The arrow ends represent positive regulation, and the black circle ends represent negative regulation. IM; inner membrane, OM; outer membrane, VPS; *Vibrio* polysaccharide, Pof; polar flagella, Laf; lateral flagella, X (in red); unknown protein for collagen or collagen peptides.

Table S1 spectrum.02992-26-s0008.docx Strains and plasmids used in this study.

ASM does not own the copyrights to Supplemental Material that may be linked to, or accessed through, an article. The authors have granted ASM a non-exclusive, world-wide license to publish the Supplemental Material files. Please contact the corresponding author directly for reuse.
