# Supplementary material for: Requirements for swarming ability by lateral flagella on an agar surface in marine Vibrio cells
Source: Microbiol Spectr. 2026 Oct 15;14(11):e02992-26. doi: 10.1128/spectrum.02992-26 (PMC13729211; doi:10.1128/spectrum.02992-26)
Supplement: Supplementary file 1 [file spectrum.02992-26-s0008.docx]

Table S1. Strains and plasmids used in this study.

| Strain or plasmid | Description | Source or reference |
| --- | --- | --- |
| *V. alginolyticus* strains | | |
| 138-2 | wild-type (Pof^+^, Laf^+^) | (1) |
| VIO5 | Rif^r^ Pof^+^ Laf^−^ | (2) |
| YM19 | 138-2 Pof^-^, Laf^+^ | (1) |
| NMB342 | YM19Δ^laf^*fliL* | (3) |
| NMB338 | 138-2 Δ^pof^*fliL* | (3) |
| NMB339 | 138-2 Δ^laf^*fliL* | (3) |
| NMB340 | 138-2 Δ^pof^*fliL*Δ^laf^*fliL* | (3) |
| Plasmids | | |
| pBAD33 | Cm^r^ P*BAD* | (4) |
| pZSW91 | pBAD33- *his6-*^laf^*fliL* | (3) |

Cm^r^, chloramphenicol resistant; Pof^+^, normal polar flagellar formation; Laf^+^, normal lateral flagellar formation; Pof^-^, defective in polar flagellar formation; Laf^-^, defective in lateral flagellar formation;^pof^*fliL*, polar flagella *fliL*; ^laf^*fliL*, lateral flagella *fliL*; P*BAD*, arabinose promoter.

1.	I. Kawagishi, Y. Maekawa, T. Atsumi, M. Homma, Y. Imae, Isolation of the polar and lateral flagellum-defective mutants in *Vibrio alginolyticus* and identification of their flagellar driving energy sources. *J Bacteriol* **177**, 5158-5160 (1995).

2.	I. Okunishi, I. Kawagishi, M. Homma, Cloning and characterization of *motY*, a gene coding for a component of the sodium-driven flagellar motor in *Vibrio alginolyticus*. *J. Bacteriol.* **178**, 2409-2415 (1996).

3.	N. Takekawa *et al.*, Structure of *Vibrio* FliL, a new stomatin-like protein that assists the bacterial flagellar motor function. *mBio* **10**, e00292-00219 (2019).

4.	L. M. Guzman, D. Belin, M. J. Carson, J. Beckwith, Tight regulation, modulation, and high-level expression by vectors containing the arabinose pBAD promoter. *J. Bacteriol.* **177**, 4121-4130 (1995).
